# Detection of PIP_2_ distributions in biological membranes using a peptide-based sensor

**DOI:** 10.1016/j.jbc.2025.110826

**Published:** 2025-10-16

**Authors:** Vinay K. Menon, Joy Wu, Alex J. Alonzo, Kevin L. Scrudders, Kaitlyn A. Rogers, Suriya Selvarajan, Andrew Walke, Jennifer Pellicier-Caraballo, Rajasree Kundu, Samsuzzoha Mondal, Ankona Datta, Shalini T. Low-Nam

**Affiliations:** 1 James Tarpo Jr. and Margaret Tarpo Department of Chemistry, Purdue University, West Lafayette, Indiana, USA; 2 Purdue University Interdisciplinary Life Sciences (PULSe) Program, West Lafayette, Indiana, USA; 3 Purdue University Department of Physics and Astronomy, West Lafayette, Indiana, USA; 4 University of Puerto Rico at Mayagüez, Mayagüez, Puerto Rico; 5 Department of Chemical Sciences, Tata Institute of Fundamental Research, Mumbai, India; 6 SLAC National Accelerator Laboratory, Stanford University, Menlo Park, California, USA

**Keywords:** phosphoinositide, membrane biophysics, membrane reconstitution, Ras protein, fluorescence, peptide-based sensor

## Abstract

Organization and composition of the plasma membrane are important modulators of many cellular programs. Phosphatidylinositol phosphate (PIP) lipids are low-abundance membrane constituents with different arrangements of phosphate groups around an inositol head group, regulating many signaling pathways. Numerous strategies have been developed to detect and track PIP species to monitor their clustering, mobility, and interactions with binding partners. We implement a peptide-based, ratiometric sensor for the detection of PI(4,5)P_2_ lipids in reconstituted membrane systems that permit absolute quantification of PI(4,5)P_2_ densities down to physiological levels less than 4 mol per cent. The sensor is membrane-permeable and easily transferable to measurements in living cells. Application of calibrated sensors to cells expressing common mutations in the small GTPase, Ras, showed a reshaping of surface PI(4,5)P_2_ levels and distributions in a mutation-specific manner. Brief treatment of G12C mutant Ras cells with the specific inhibitor, Sotorasib, resulted in alterations to surface PI(4,5)P_2_ arrangements that resemble the wild-type (WT) Ras. Thus, the rapid redistribution of PI(4,5)P_2_ lipids upon drug treatment emphasizes the tight coupling between membrane composition, organization, and downstream signaling outcomes. Tools and strategies to monitor membrane composition alongside cellular behaviors could provide pipelines to characterize therapeutics and improve the mechanistic understanding of how protein-lipid coupling drives cellular programs.

Despite very low abundance in the plasma membrane, phosphatidylinositol phosphate (PIP) lipids generate specific signaling outcomes ranging from actin reorganization to immune signaling (1, 2, 3, 4, 5). PIP lipid species, defined by unique arrangements of phosphate groups around an inositol head group, predominate the cytoplasmic leaflet of the plasma membrane and comprise approximately 2 mol percent of the total lipid content, based on lipidomic data (6). Phosphatidylinositol-4,5-bisphosphate (PI(4,5)P_2_) is the most abundant PIP species and plays a key role in the activation of the guanine nucleotide exchange factor (GEF) son of sevenless (SOS) through pleckstrin homology (PH) domain-mediated release of autoinhibition (7, 8). Active SOS primes the activation of the small GTPase, Ras, and drives mitogenic signaling.

Ras mutations are common drivers in many cancers which has led to extensive efforts to block oncogenic Ras signaling. The recent development of inhibitors specific for KRas4B allele-specific mutations has provided a major advance in several cancer therapies. One such inhibitor, Sotorasib, is a selective covalent inhibitor targeting the KRas4B G12C mutation commonly found in colorectal and lung cancers (9, 10, 11). Impacts on the treatment of non-small cell lung cancers have been promising with a positive tolerability profile. Mechanistically, irreversible binding to the GDP-bound, inactive form of KRas4B G12C results in diminished ERK phosphorylation and decreased cell proliferation (12). Thus, Ras activities at the plasma membrane emphasizes how protein and lipid organization can prime downstream signaling and cellular behavioral outcomes.

As a substrate, PI(4,5)P_2_ is cleaved into second messengers inositol triphosphate (IP_3_) and diacylglycerol (DAG) by phospholipase C, and is converted to PI(3,4,5)P_3_ by PI3K (13, 14, 15, 16). PI(3,4,5)P_3_ activates downstream signaling pathways involved in cell growth and survival, and spatial segregation of PI(4,5)P_2_ and PI(3,4,5)P_3_ is a feature of front-rear specification during cell migration (17, 18, 19, 20). Thus, PI(4,5)P_2_ functions as an activator for many signaling programs but, in parallel, it is depleted by competing processes. Adding to the complex dynamics in the MAP kinase pathway, Ras itself interacts with PI(4,5)P_2_ and recruits PI3K to the surface to promote migratory programs (16, 21). Given the dependence of many signaling programs on PI(4,5)P_2_ abundance, the detection of this rare lipid and its transformations has been the subject of great interest.

Heterogeneity in PI(4,5)P_2_ distribution has been a hallmark of cellular mechanisms, such as crawling or endocytosis, whose collective processes span the nanometer-to-micron length scale and seconds-to-minutes time scale (1, 2, 3, 4, 5, 22). Measurements of PI(4,5)P_2_ mobility and dynamics have mainly been based on fluorescently-tagged binding domains or antibody-based detection. The PH domain from phospholipase C delta 1 (PH-PLCδ1) is commonly used to track PI(4,5)P_2_ mobility and has shown both rapid and slow diffusive states (23). Restricted diffusion has been implicated in PI(4,5)P_2_ signaling and emphasizes how local enhancement in PI(4,5)P_2_ may overcome the overall low abundance (24). Recent work using the C-terminal domain of Tubby showed modest immobilization of PI(4,5)P_2_ in the plasma membrane. This work highlighted that mobility based on detection using PH-PLCδ1 exhibits slower diffusion of PI(4,5)P_2_ by an unknown interaction that may confound the interpretation of the data. Other challenges in detecting PI(4,5)P_2_ using these domain-based biosensors include their relatively large sizes and the requirement for microinjection, electroporation, or transfection into cells for visualization (25). Thus, characterization of PI(4,5)P_2_ distributions and dynamics across a population of cells may be impaired by the technical challenges in using these biosensors.

Datta and colleagues recently introduced a cell-penetrating, ratiometric, peptide-based PI(4,5)P_2_ sensor, DAN13aa (25). The short, cationic sequence, derived from the actin-binding protein gelsolin, penetrates bilayer membranes and is selective for PI(4,5)P_2_ (25). To calibrate the DAN13aa biosensor, we developed an *in vitro* reconstitution-based approach using stacked supported lipid bilayers (SSLBs) across a range of PI(4,5)P_2_ densities, including the low levels that approach predicted physiological densities (6). Fast sensor binding kinetics and a linear responsiveness of the sensor permitted characterization of PI(4,5)P_2_ distributions in living cells expressing point mutations of KRas4B in an isogenic mouse embryonic fibroblast (MEF) background. Focusing on common KRas4B mutations with impaired GTPase activity that exacerbate PI3K activation (26), an unexpected heterogeneity emerged. Both levels of PI(4,5)P_2_ and organization at the membrane were altered at steady state. Notably, the densities PI(4,5)P_2_ and their distributions, characterized by a heterogeneity metric, were suggestive of fingerprints for the different G12 or G13 mutations. We propose that an increase in spatial heterogeneity of phosphoinositide distribution is a hallmark of oncogenesis and may provide a biomarker for disease state and therapeutic efficacy.

## Results

### Stacked supported lipid bilayers (SSLB) prevent DAN13aa substrate sticking

**Figure 1:**
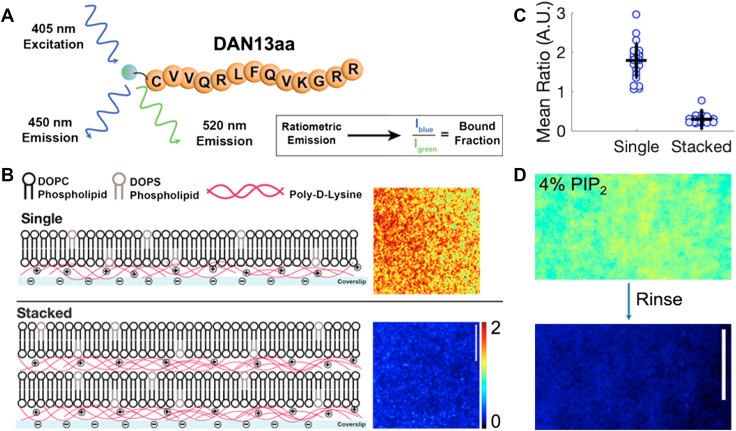
**DAN13aa sensor sensitively detects PI(4,5)P_2_ in stacked supported lipid bilayers.***A*, the DAN13aa sensor is detected ratiometrically using 405 nm excitation and emission at 450 nm and 520 nm. *B*, schematics of single (*top*) and stacked SLBs (*bottom*) on poly-lysine-coated glass substrates. In each case, the ratio of emissions is shown for a representative field of view. *C*, quantification of mean ratio in single and stacked SLB configurations for at least two replicates and multiple fields of view. *D*, ratiometric signal for a 4% PIP2-containing top bilayer in a stacked configuration before (*top*) and after (*bottom*) rinsing with 1x PBS buffer. Color scale shown in (*B*) are equivalent to that of (*D*). All scale bars are 10 μm.

During its development, the DAN13aa sensor was shown to have selectivity for PI(4,5)P_2_ over PI(4)P and a micromolar affinity (25). Prior work with the sensor measured features throughout the cell volume *via* confocal and 2-photon imaging (25). For this work, we focused on the plasma membrane to emphasize signaling onset at the surface. The sensor is ideally suited to PI(4,5)P_2_ detection in cellular membranes with a strong sensitivity to the physiologically low levels of the lipid in the plasma membrane. To monitor sensor responsiveness at PI(4,5)P_2_ densities ranging from 1 to 10 mol percent, we developed an *in vitro* reconstituted platform based on supported lipid bilayers (SLBs) and implemented surface-selective imaging in a total internal reflection (TIR) fluorescence configuration. Measurements were made with the sensor maintained in solution to establish a steady state of exchange between the membrane and the bulk and to serve as the model for experiments in cells where the sensor will be present in the cytoplasm and engage PI(4,5)P_2_ in different cellular membranes. The cationic 13-mer peptide is conjugated to the solvatochromic fluorophore, 2-dimethylamino-6-acyl-naphthalene (DAN) at the N-terminal cysteine residue. Following excitation at 405 nm, dual wavelength detection was used to monitor the shift from green emission in an aqueous environment (unbound, λ_em_max_ = 520 nm) to blue emission in a hydrophobic environment (bound, λ_em_max_ = 450 nm; Fig. 1*A* (25)). Data were represented as a bound fraction based on the ratio of bound: unbound signal.

In initial measurements, PI(4,5)P_2_-containing SLBs were formed by vesicle fusion on a negatively charged solid glass support. Consistent with a strong membrane permeability, the positively-charged DAN13aa sensor passed easily through SLBs and stuck non-specifically to the underlying glass substrate, even in the absence of PI(4,5)P_2_ (Fig. 1*B*, top; Fig. S1). Thus, single, planar SLBs were insufficient to calibrate the DAN13aa sensor response curve.

Using a previously described strategy, we introduce passivated, stacked SLBs (SSLBs) as a reconstituted membrane platform (27). The stacked SLBs consisted of two supported lipid bilayers deposited alternately on top of layers of poly-D-lysine (Fig. 1*B*). The bottom bilayer was composed of DOPC and DOPS lipids that were shown to have negligible interactions with the sensor (25). The upper bilayer could be doped with PI(4,5)P_2_ lipids as needed. The stacked SLB platform ensured that the cationic poly-lysine repelled the positively charged DAN13aa sensor and resulted in a dramatic reduction in background in the absence of PI(4,5)P_2_ in the bilayers (Fig. 1, *B* and *C*). Subsequent *in vitro* experiments were conducted using SSLBs and cell data were collected on glass substrates coated with poly-lysine.

Mobility of each of the bilayers in the SSLBs was confirmed by fluorescence recovery after photobleaching (FRAP; Fig. S2). Single lipid mobility in the upper bilayer was also verified through the introduction of 1 mol percent of fluorescently-tagged PI(4,5)P_2_. Following photobleaching to achieve single-molecule densities, individual lipids were easily detected diffusing laterally in the upper bilayer (Fig. S3). Prior observations had identified some decreased mobility in PI(4,5)P_2_-containing single SLBs due to lipid flipping to the lower leaflet and strong hydrogen bonding with the glass substrate (28). Notably, the second poly-D-lysine layer was found to be mobile, presumably due to the fluidity of the underlying SLB (Fig. S2). Thus, the biophysical characteristics of the SSLB configuration maximized our ability to quantify DAN13aa sensor-based detection.

The application of the DAN13aa sensor to PI(4,5)P_2_ dynamics should minimize perturbation to normal processes at the membrane. Competition for PI(4,5)P_2_ binding or long interaction lifetimes could alter normal signaling processes. Therefore, we verified rapid exchange of DAN13aa sensor on SSLBs with 4 mol percent PI(4,5)P_2_. The measured bound fraction rapidly returned to background levels following a quick rinse with buffer, suggesting fast binding and unbinding kinetics (Fig. 1*D*). This is consistent with the ones of micromolar dissociation constants that were reported for the sensor (25).

### SSLBs enable precise calibration of the DAN13aa PI(4,5)P2 biosensor

**Figure 2:**
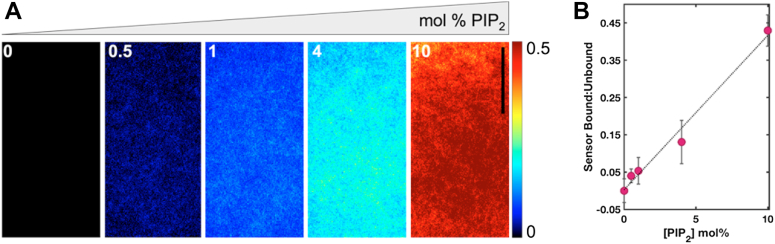
**Calibration of DAN13aa in stacked supported lipid bilayers (SSLBs).***A*, SSLBs were constructed with 1 mol% DOPS/99 mol% DOPC in the bottom bilayer. The top bilayer contained either 1 mol% DOPS/99 mol% DOPC; 0.5 mol% PI(4,5)P_2_/99.5 mol% DOPC; 1 mol% PI(4,5)P_2_/99 mol% DOPC; 4 mol% PI(4,5)P_2_/96 mol% DOPC; or 10 mol% PI(4,5)P_2_/90 mol% DOPC as indicated. Ratiometric signal from 405 nm excitation and emission collected at 405 or 488 nm is shown. Units are arbitrary. *B*, the ratiometric intensity of 405 to 405 to 405 to 488, corresponding to the bound fraction of DAN13aa, was plotted as a function of PI(4,5)P_2_ mol% in the top bilayer and fit with a linear regression. The linear regression line is y = 0.041x + 0.002 with R^2^ = 0.964. Scale bar = 10 μm.

PI(4,5)P_2_ densities in cellular membranes have been estimated to be approximately 20,000 molecules/μm^2^ and 8 × 10^6^ - 6.8 × 10^7^ molecules in the inner leaflet of mammalian cells (29, 30, 31, 32, 33). Calibration of the DAN13aa biosensor in SSLBs ranging in PI(4,5)P_2_ densities from 0 to 10 mol percent spanned this range (Fig. 2*A*). We confirmed the phosphate composition in the small unilamellar vesicles used in the formation of SSLBs by a phosphate quantification assay. Digestion of lipid mixtures by perchloric acid resulted in free phosphates that were quantified by a colorimetric reaction with a molybdenum-containing compound (34). Using vesicles containing 0, 1, and 4 mol percent porcine brain PI(4,5)P_2_, we showed that the phosphate levels were tightly confined to the expected amounts of the phosphoinositide (Fig. S4).

There was a very strong linear correlation (R^2^ = 0.964) between the PI(4,5)P_2_ content in the stacked bilayer and the corresponding ratiometric signal intensity from the DAN13aa biosensor (Fig. 2*B*). A typical challenge in designing phosphoinositide sensors is the high degree of structural similarity between the lipids. Given the role of Ras in activating PI3K to convert PI(4,5)P_2_ into PI(3,4,5)P_3_, we measured the ability of DAN13aa to distinguish between these phosphoinositides. Despite some promiscuity in sensor detection of PI(3,4,5)P_3_-containing SSLBs, the DAN13aa sensor detects PI(4,5)P_2_ with 2-3-fold selectivity over PI(3,4,5)P_3_ (Fig. S5). Thus, in cells, the application of DAN13aa to PI(4,5)P_2_ density is predominated by the dually phosphorylated lipid but may detect contributions from PI(3,4,5)P_3_. We predict this occurs, in particular, when local PI(4,5)P_2_ is converted to PI(3,4,5)P_3_ by PI3K and the sensor is already at the membrane. We also checked the extent of detection of the lipids phosphatidylinositol 4-phosphate (PI(4)P) and phosphatidylserine (PS), another anionic lipid that is often abundant in cell membranes. Similar to PI(3,4,5)P_3_, there was some detection of these other lipids, but with 2- to 3-fold selective preference for PI(4,5)P_2_ (Fig. S5). Thus, some non-specific binding to negatively charged lipids occurs when using DAN13aa but is predominated by *bona fide* PI(4,5)P_2_ lipids. A recently reported PI(3,4,5)P_3_ sensor has a strong selectivity over PI(4,5)P_2_ and might enable dual detection of these lipids (35). Further application of the sensor to living cells is promoted by a negligible difference in sensor sensitivity at room temperature as compared to 37 °C (Fig. S6).

### Application of the DAN13aa sensor to steady state PI(4,5)P2 levels and distributions in living cells

**Figure 3:**
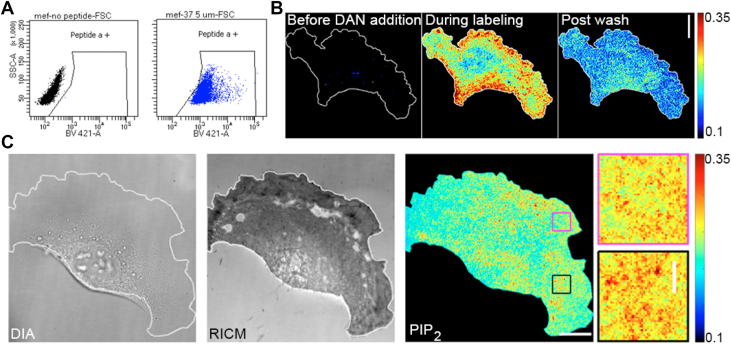
**Labeling of cells with DAN13aa sensor.***A*, Flow cytometry of WT MEF cells without (*left*) and with (*right*) DAN13aa sensor. *B*. Labeling of WT MEF cells before (*left*) and during (*middle*) DAN13aa labeling. Following a rinse with buffer the steady state DAN13aa ratiometric signal was monitored (*right*). During analysis, raw images are background corrected to remove contaminating signals due to cellular biomolecules that are responsive to 405 nm illumination. *C*, WT MEF cells showing diascopic image, adherent membrane imaged in interference contrast microscopy (IRM) and PI(4,5)P_2_ density following adjustment of ratiometric signal using calibration curve. Regions of interest are enlarged to the *right*. All scale bars are 10 μm. Colorbars are in units of mole percent.

For experiments in cells, we used the isogenic KRas4B cell lines established in the MEF background (26). The cells are NRas and HRas null and include either wild-type (WT) KRas4B or common clinical Ras mutants. These cells provide a common background to measure the distribution of PI(4,5)P_2_ lipids in the plasma membrane under modulation by oncogenic Ras. Initial characterization of the sensor in cells was carried out using the WT cells as a model. Consistent with the high degree of membrane permeability designed into the cationic DAN13aa peptide, sensor uptake, measured by flow cytometry, was uniform across the cell population (Fig. 3*A*). Thus, the sensor enables measurement of PI(4,5)P_2_ features in a heterogeneous population of cells and without the need for expression or introduction of an exogenous biosensor.

Labeling of cells was also visualized using ratiometric imaging in TIRF to detect PI(4,5)P_2_ lipids at the adherent membrane. The sensor was rapidly taken up by cells in minutes and reached a high level of signal. Following a short incubation and a rinse with buffer, cells retained an equilibrium amount of sensor and showed a measurable ratiometric signal (Fig. 3, *B* and *C*). Using the calibration from the SSLBs, the measured PI(4,5)P_2_ levels were converted to mole percentages. Overall, PI(4,5)P_2_ distributions in WT cells appeared to be uniform but some clustered lipids with local concentrations up to almost 1 mol percent were observed (Fig. 3*C*). Small clusters of PI(4,5)P_2_ lipids are consistent with previous observations (36, 37). We confirmed the distribution of PI(4,5)P_2_ lipids using immunofluorescent detection, which also showed some local clustering but a spread across the membrane (Fig. S7).

The comparison in ratiometric detection of PI(4,5)P_2_ using the DAN13aa probe was made to a very common PH-domain-based binding to PI(4,5)P_2_ headgroups. We used the PH domain of PLCδ1 tagged with a red fluorescent protein and expressed in WT MEF cells. PI(4,5)P_2_ detection by PH domain association is the most common tool to measure surface distributions and mobility at the surface (23, 24, 38, 39). Cells expressing the PH construct were co-labeled with the DAN13aa sensor and each signal was detected in TIRF. Prior characterization of the DAN13aa sensor suggested that each probe could access different pools of PI(4,5)P_2_ within the cellular volume (25). Our direct detection in single cells confirmed that the labels could be applied simultaneously to detect PI(4,5)P_2_ at the plasma membrane. The mean intensity level of the DAN13aa sensor signals reflected a much narrower distribution than the PH domain (Fig. S8), which may indicate features of the PH domain signal that are dominated by the expression of the transfected sensor. The signal amplitudes from the sensors were poorly correlated in a cell-to-cell manner.

**Figure 4:**
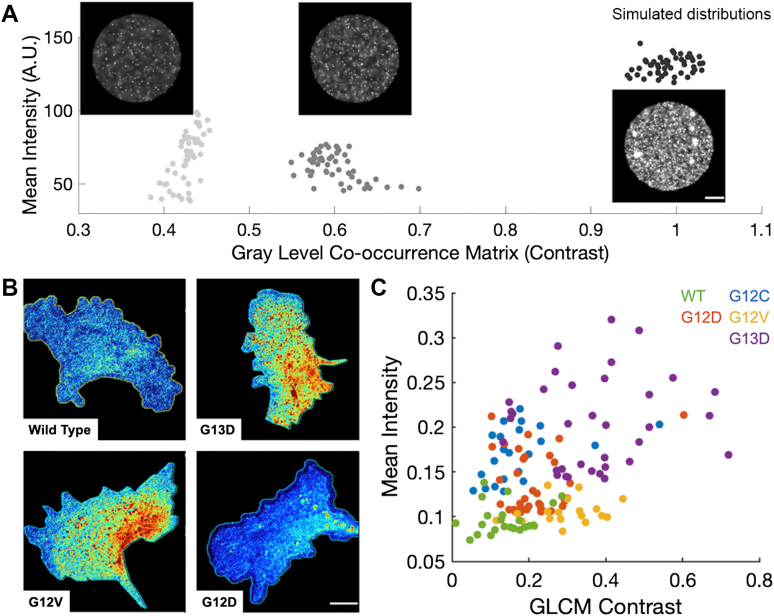
**Clustering of PI(4,5)P_2_ densities in single Ras mutant MEFs.***A*, simulations of PI(4,5)P_2_ distributions. Cell surface is simulated as circle and each simulated, Gaussian spot is a PI(4,5)P_2_ molecule. Three sets of simulated PI(4,5)P_2_ densities and distributions are shown with a representative raw image and the corresponding single cell data as individual points, shown in grayscale. The most heterogeneous data (high contrast) are to the right with simulated PI(4,5)P_2_ clusters. *B*, representative images of MEF Kras4B cells labeled with DAN13aa and imaged at steady state. *C*, texture analysis of MEF cells labeled with DAN13aa and visualized in TIRF. Data are shown as mean intensity (in mole percent) *versus* texture value (GLCM Contrast; a.u.) per cell. Data from three independent experiments. All scale bars are 10 microns.

To assess the information contained in each dataset, we applied texture analysis based on the Gray Level Co-occurrence Matrix (GLCM) to quantify the features of the data for each masked cell (40). In the GLCM approach, features of neighboring pixels are extracted to assess the signal heterogeneity. GLCM analysis extracts the distribution of co-occurring pixel grayscale values across an image to show patterns and gradients within an image (41, 42). Although the data from across the cell is reduced to a single, representative parameter, this measure provides spatial features across the entire cell and not simply localized to specific subregions. To test the application of GLCM analysis to lipid distribution data, we generated a series of simulated datasets with spots to represent individual PI(4,5)P_2_ molecules across a mock cell surface (Fig. 4*A*). The simulations included variations in the amount of PI(4,5)P_2,_ which is reflected in the mean intensity parameter and different probabilities of lipid clustering. Differences in local PI(4,5)P_2_ density were quantified using the GLCM contrast parameter, where larger values reflect an increase in heterogeneity across the surface (Fig. 4*A*). Larger PI(4,5)P_2_ clusters across the surface were consistent with an increased GLCM heterogeneity (Fig. 4*A*).

Using the GLCM contrast parameter, we found that the assessment of surface PI(4,5)P_2_ distributions in WT MEF cells was different when using the PH domain or the DAN13aa sensor (Fig. S8). Again, the overall distribution in the contrast parameter was broader in the PH data. Taken together, the DAN13aa sensor reflects less cell-to-cell variation than detection with the PH domain sensor, which may reflect altered PI(4,5)P_2_ diffusion that has been reported for the latter (24). The differences in the PI(4,5)P_2_ detection may also reflect underlying competition with other macromolecules binding the phosphoinositide at the plasma membrane. Given the low, micromolar affinity of the DAN13aa sensor and fast unbinding we measured, we predict that this competition is low.

### Steady state PI(4,5)P2 densities in MEFs expressing Ras mutants

To extend the cellular observations to MEFs expressing KRas mutants, we selected the G12 and G13 mutation hotspots to focus on the mechanism of slowed Ras inactivation *via* interference of GTPase accelerating protein (GAP) binding (43). The MEF background was previously established to enable single mutational studies in an isogenic background (26). To avoid toxicity from extended exposure to 405 nm light in the ratiometric imaging, PI(4,5)P_2_ surface levels and distributions were monitored under steady state conditions. Observations of single cells expressing WT, G12C, G12D, G12V, and G13D Ras genotypes, showed that the average PI(4,5)P_2_ levels covered a narrow range of less than 1 mol percent, but were different for each genotype (Fig. 4, *B* and *C*). Remarkably, when compared to WT cells, the mutant Ras MEF cells had greater heterogeneity in PI(4,5)P_2_ densities. This observation is inconsistent with a simple explanation that hyperactive Ras primes conversion of PI(4,5)P_2_ to PI(3,4,5)P_3_ by PI3K to promote leading edge protrusivity. Our measurements were made under tonic signaling conditions which may have obscured some excitable activities that have been observed in Ras, PI3K, and actin networks (44).

We questioned the ability of PI(4,5)P_2_ densities and distributions to serve as a fingerprint for the various Ras mutations. When the mean PI(4,5)P_2_ intensities were plotted against the GLCM values, the mutant genotypes were found to cluster into characteristic regions of the resulting density-spatial heterogeneity space (Fig. 4*C*). G13D cells were found to have the highest levels of PI(4,5)P_2_ and degree of heterogeneity, and WT cells had the lowest PI(4,5)P_2_ densities and most homogeneous distributions. The findings in the WT cells are consistent with a tight coupling between PI(4,5)P_2_ levels and cellular signaling for homeostasis. The G12D and G12V cells had similar mean PI(4,5)P_2_ levels, but the latter had a slightly higher degree of heterogeneity in distribution. Thus, alterations in Ras activities through the G12 and G13 mutations are reflected in the surface organization of the plasma membrane.

**Figure 5:**
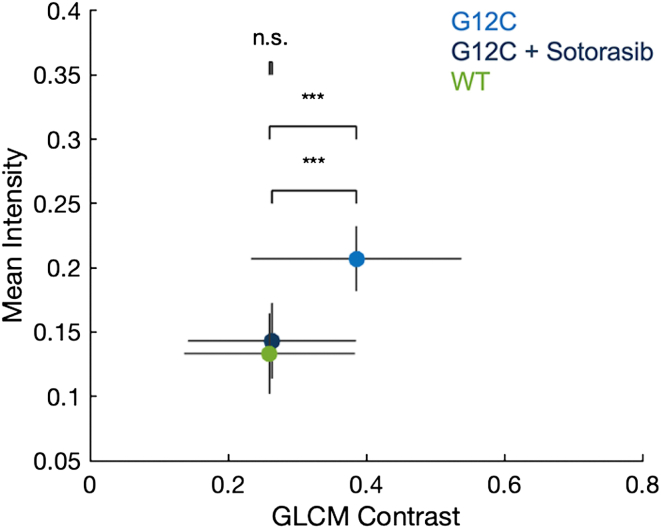
**Treatment of MEF cells expressing the KRas4B G12C Ras mutant with Sotorasib leads to a PIP_2_ surface abundance and distribution that resembles the WT KRas4B protein.** Data are plotted as mean and standard deviation for mean intensity in mole percent and GLCM contrast (a.u.). Data are from three independent experiments and include at least 40 cells for each condition. Significance by Wilcoxon rank sum test for the GLCM contrast. ‘∗∗∗’ is *p* < 0.01 and ‘n.s.’ is not significant.

Given our finding that mutant genotypes segregated in the density-spatial heterogeneity space, we set out to understand if KRas4B inhibitors could be profiled using the surface PI(4,5)P_2_ distribution. Treatment of Ras-specific cancers with small molecule inhibitors has generated clinical efficacy, particularly the G12C inhibitors Sotorasib (AMG 510; (12, 45)) and Adagrasib (MRTX849; (46)). Mechanistically, these inhibitors covalently bind the mutant cysteine residue in the inactive, GDP-bound form of KRas4B and block its activation. Following treatment with 20 nM Sotorasib, the mean PI(4,5)P_2_ levels and heterogeneity in G12C mutant cells resembled the WT cells (Fig. 5*A*). Thus, treatment not only blocked G12C Ras activity but also enabled global reorganization of the cell surface. Remarkably, these dramatic effects were observed after only 1 h of pretreatment with the drug. These observations are consistent with the strong impact of PI(4,5)P_2_ behaviors on many functional decisions in a variety of cells.

## Discussion

Establishment of PIP heterogeneity is a hallmark of several cellular processes. The spatial heterogeneity spans several orders of magnitude from signaling and trafficking events to cell-wide redistribution (1, 2, 3, 17, 47). The extent to which cellular signaling programs are sensitive to heterogeneities in local PI(4,5)P_2_ densities has been speculated but remains incompletely explored. The typical diffusion coefficient for PI(4,5)P_2_ is reported around 0.1 to 0.3 microns squared per second with individual lipids detected to sample fast and slow mobility states (24, 48). It is possible that clustering of PI(4,5)P_2_ enhances some signaling activities or that high densities of some proteins, especially those that are membrane-anchored, could confine the lipids themselves (13, 24). Membrane PI(4,5)P_2_ hotspots could, alternatively, be a site for conversion to PI(3,4,5)P_3_ as expected in Ras-driven membrane recruitment and activation of PI3K.

To provide a rapid assessment of the levels and arrangements of PI(4,5)P_2_ at the plasma membrane, we sought to apply a recently introduced peptide-based sensor to the quantification of PI(4,5)P_2_ densities at the low levels expected for biological systems. We coupled the DAN13aa sensor with a reconstituted, stacked supported lipid bilayer system to show that the ratiometric signal scales linearly with the lipid density. The fast-binding kinetics are expected to be minimally perturbative to PI(4,5)P_2_-dependent processes. Further, the utility of a calibration curve is bolstered by the facile application of the sensor to populations of living cells due to its high membrane permeability.

Our observation of PI(4,5)P_2_ heterogeneity is consistent with a major role in generating signaling outcomes similar to reports of enrichment in PI(3,4)P_2_ in response to platelet-derived growth factor (PDGF) stimulation (49). Thus, the ability of a cell to regulate PI(4,5)P_2_ levels and arrangements may hint at a broader design principle. Careful identification of lipid fingerprints and their contributions to cellular activities is highly desirable. The slight promiscuity of the biosensor with other lipids we tested could suggest that some of the heterogeneity we measured is due to a locally high concentration of non-specific anionic lipids. Given the enhanced sensitivity of DAN13aa for PI(4,5)P_2,_ we predict that this lipid predominates these regions. Most importantly, our measurements provide an upper bound to the PI(4,5)P_2_ density on the plasma membrane, emphasizing that the small mole percentage levels of this lipid are highly consequential to cell-wide outcomes.

When considering specific Ras mutations in an isogenic line, we found a surprising pattern to PI(4,5)P_2_ spatial distributions and mean PI(4,5)P_2_ levels. The application of the DAN13aa sensor could distinguish between the mutants without a requirement for super-resolution of lipid organization. High PI(4,5)P_2_ densities in G13D mutant cells as compared to G12 mutants may suggest enhanced PTEN-based conversion of PI(3,4,5)P_3_ to PI(4,5)P_2_. This agrees with data showing that elevated PI3K activity is opposed by PTEN phosphatase (49). The levels and heterogeneity of mutant cells is notably shifted from wild-type cells, which may indicate that changes in PI(4,5)P_2_ regulation at the membrane is a hallmark of Ras mutant cells that can be readily profiled. This is exemplified by the discovery that the KRas4B G12C inhibitor, Sotorasib, not only generates cell surfaces that resemble WT cells but, more surprisingly, that this occurs very rapidly. Thus, the sensor also provides access to population profiling over fast timescales even in the absence of continuous ratiometric imaging. New opportunities are possible with variants of the sensor that also enable increased spatial and temporal resolution through alternate fluorophores. In particular, achieving single-molecule resolution could provide access to the on- and off-rate of sensor binding and enable simultaneous imaging with other proteins to monitor dynamic interplay between phosphoinositide conversion, signaling, and other programs.

Increased PI(4,5)P_2_ density is expected to enhance many signaling pathways, in addition to serving as a substrate for conversion to PI(3,4,5)P_3_, as discussed above. How these competing programs are affected by spatial heterogeneity in PI(4,5)P_2_ is unknown. We speculate that this may be a key source of regulating and partitioning cellular activities. Local increases in PI(4,5)P_2_ density would be expected to drive activation of signaling from membrane condensates such as LAT assemblies in T cells and proposed EGFR assemblies in epithelial cells through SOS activation and priming MAPK signaling (7, 50, 51). Whether PI(4,5)P_2_ is sequestered within these assemblies or can be hydrolyzed to drive disassembly, the role for these rare lipids may be highly consequential. The global modulation of PI(4,5)P_2_ demonstrated here suggests that the composition of the membrane will be a key indicator of cell state and could have diagnostic value.

## Experimental procedures

### Reagents

Sotorasib (Selleck Chemicals) was preincubated with cells at a 20 nM concentration for 1 h in an incubator. The DAN13aa sensor was produced and purified according to previously established protocols (25). The PH domain of PLCδ1, tagged with RFP, was a gift from the Stahelin Lab (Purdue University).

### Cell culture and transfection

All cells were maintained at 37 °C and 5% CO_2_. Isogenic MEFs were cultured as described previously (26). Briefly, cells were cultured in phenol-free, high-glucose Dulbecco’s Modified Eagle Medium (DMEM) supplemented with 10% (v/v) fetal bovine serum (FBS), 100 IU/ml penicillin, 100 μg/ml streptomycin, 2 mM L-glutamine, and 1 mM sodium pyruvate. For transfection with the PH domain, MEF cells plated to target one million cells at the time of transfection. Transfection was performed with Lipofectamine 2000 (Invitrogen) as the carrier material. A range of 1 to 3 μg of plasmid was incubated with 6 μl of Lipofectamine 2000 in reduced serum medium (Opti-MEM, Gibco) for 5 min to form DNA/polymer complexes. The complexes were added dropwise to cells in fresh DMEM. After approximately 24 h, the media was exchanged once more to fresh DMEM media and were ready to be imaged.

### Preparation of small unilamellar vesicles (SUVs) and imaging chamber assembly

On the day of experiments small unilamellar vesicles (SUVs) with the following composition were formed *via* tip sonication using lipids obtained from Avanti Polar Lipids (Alabaster, AL).

**Table:** 

Lipid composition of small unilamellar vesicles			
Sample	1,2-Dioleoyl-sn-glycero-3-phosphocholine (DOPC)	1,2-Dioleoyl-sn-glycero-3-phospho-L-serine (DOPS)	L-α-phosphatidylinositol-4,5-bisphosphate (PIP_2_)
0% PIP_2_	99 mol%	1 mol%	0 mol%
0.5% PIP_2_	99.5 mol%	0 mol%	0.5 mol%
1% PIP_2_	99 mol%	0 mol%	1 mol%
4% PIP_2_	96 mol%	0 mol%	4 mol%
10% PIP_2_	90 mol%	0 mol%	10 mol%

Following sonication, the lipids were centrifuged for 30 to 60 min at ∼23,000*g* at 4 °C. The resulting supernatant was then transferred to a fresh tube to avoid potential contamination from the titanium pellet. Lipids were stored on ice until needed.

25 mm #1.5 coverslips (Warner Instruments, Holliston, MA) were sonicated for 30 min in 50:50 isopropyl alcohol:ddH_2_O, and then rinsed thoroughly with ddH_2_O. The coverslips were then etched for 5 min with freshly prepared piranha solution (3:1 H_2_SO_4_:H_2_O_2_) and rinsed thoroughly with ddH_2_O. The coverslips were then dried with N_2_ and assembled into an Attofluor Cell Chamber (Invitrogen, Waltham, MA).

### Stacked supported lipid bilayers (SSLB)

Construction of stacked supported lipid bilayers (SSLB) was adapted from (27) and is shown schematically in Figure 1*B*. Assembled imaging chambers were coated with 0.1 mg/ml poly-D-lysine (PDL, Gibco) and rinsed 3 × 5 ml with 1X PBS. This was followed by one final rinse with 10X PBS. 750 ml was removed and 250 ml of freshly prepared 0 mol% PIP_2_ SUVs in aqueous suspension was added to the chamber resulting in a final lipid concentration of 0.5 mg/ml. Using a Pasteur pipette, one drop (∼20 μl) of 6M HCl was added to each chamber and incubated at room temperature for 4 min. The increased salt concentration and lower pH was shown to be required to drive vesicle fusion and form a contiguous bilayer (data not shown). The chamber was then rinsed 3 × 5 ml 1X PBS. The next layer of PDL was diluted 1:10 into the chamber prior to rinsing with 1X and 10X PBS as before. The final bilayer (0–10% PI(4,5)P_2_) was then deposited as described above. The resulting stacked bilayers were imaged immediately.

### Characterization of stacked supported lipid bilayers (SSLB)

Construction of stacked supported lipid bilayers (SSLBs) was validated in two separate dual-color experiments, one for the lipid bilayers and another for the poly-lysine layers. For these experiments, 0% PIP2 SUVs were used for both lipid bilayers. After deposition, the first lipid bilayer was stained with the green lipophilic carbocyanine dye, DiO (λ_Ex_/λ_Em_ = 484/501 nm, ThermoFisher Scientific). The sample was then imaged in TIRF on piranha-etched coverslips. Fluorescence recovery after photobleaching (FRAP) was conducted to confirm mobility of the bilayer (Fig. S2). The same process was repeated in the same sample for the top bilayer using the far-red fluorophore, DiD (λ_Ex_/λ_Em_ = 644/663 nm, ThermoFisher Scientific).

For the poly-lysine layers, a 25% (m/m) solution was created by doping either poly-L-lysine-Cy3 (λ_Ex_/λ_Em_ = 554/566, NanoCS) or poly-L-lysine-Cy5 (λ_Ex_/λ_Em_ = 647/665, NanoCS), for the bottom layer or top layer, respectively, into non-fluorescent poly-D-lysine (Gibco). The sample was then imaged in TIRF on piranha-etched coverslips. FRAP was conducted on each layer (Fig. S2).

### Calibration of DAN13aa in stacked supported lipid bilayers (SSLB)

Freshly prepared stacked supported lipid bilayers (SSLBs) with 0 to 10 mol% porcine brain PI(4,5)P2 in the top bilayer were treated with 10 nM (final concentration) of the PI(4,5)P2 peptide-based biosensor, DAN13aa, and incubated at room temperature for 10 to 15 min. Samples were then mounted on the Nikon Eclipse Ti2 microscope fitted with a room temperature stage and a 100X oil TIRF objective (numerical aperture = 1.49). Each sample was excited using a 405 nm laser in TIRF and emission was collected at 405 nm (405–405, PIP2-bound) and 488 nm (405–488, unbound). All data were presented as a ratio of I_405-405_ to I_405-488_ to represent the bound fraction of the peptide. Samples were exposed for 50 ms at 10 mW. Samples were then washed 3 × 5 ml with 1X PBS and imaged again using identical settings in a separate field of view. Pre-wash data was used to construct the calibration curve presented above.

### Flow cytometry

Wild type isogenic MEFs (KRas^+/+^, HRas^−/−^, NRas^−/−^) were diluted to the appropriate concentration in phenol-free complete media. The cells were incubated at 37 °C with DAN13aa for 10 to 15 min before vortexing and running on a BD Fortessa Cell Analyzer housed in the Purdue Flow Cytometry and Cell Separation Facility. Samples were excited with a 405 nm laser, and 10,000 independent events were collected.

### Immunofluorescence

Mouse embryonic fibroblasts G12D cells were plated onto glass coverslips pre-coated with 0.1% poly-L-lysine solution to increase cell adherence. The cells were washed three times with 1X Phosphate Buffer Saline (PBS). The cells were then fixed at room temperature for 1 min using 0.5% Paraformaldehyde (PFA) solution in PBS. Afterward, the cells were washed three times with 1X PBS. Following fixation, cells were permeabilized for 3 min using 0.2% Triton X-100 solution in PBS at room temperature. A 1% m/v solution of Bovine Serum Albumin(BSA) in PBS was used to block cells for 15 min. The Mouse Anti-PI(4,5)P_2_ (Echelon Biosciences, clone 2C11) was diluted to 10 ug/ml in the blocking solution. The primary antibody was incubated for 30 min at room temperature. Cells were then subjected to three washes with 1x PBS. The goat anti-mouse A555 (Thermo Fisher) was diluted to 10 ug/ml in the BSA solution. The secondary antibody was incubated on the cells at room temperature for 1 h. Cells were washed again three times with 1x PBS. To visualize the PIP2 antigen localization, total internal reflection fluorescence TIRF microscopy was used.

### *In cellulo* measurements of PIP2 using DAN13aa

Isogenic MEFs were plated on sterilized, poly-D-lysine coated, #1.5 glass coverslips in complete, phenol-free media 12 to 18 h prior to imaging. Coverslips were then assembled into an Attofluor Cell Chamber (Invitrogen, Waltham, MA) and mounted on the Nikon Eclipse Ti2 microscope fitted with an incubated stage (Tokai Hit; humidified, 37 °C + 5% CO_2_) and a 100X oil TIRF objective (numerical aperture = 1.49). The DAN13aa sensor was added to the chamber at 1 μM final concentration and allowed to incubate for 15 min. Each sample was excited using a 405 nm laser in TIRF, and emission was collected at 405 nm (405–405, PIP2-bound) and 488 nm (405–488, unbound). Cells were also imaged using the label-free imaging modality, reflection interference contrast microscopy (RICM).

### Phosphate quantification assay

A working stock of Ammonium Molybdate/Malachite Green (3:1 by volume 0.2% Malachite Green in MQ H_2_O:4.2% Ammonium Molybdate in 5 M HCl) was prepared and filtered and stored in the dark. SUVs of 0, 1, or 4 mol% PIP_2_ were prepared as above for bilayers. A DOPC standard curve was based on samples with a range of 1 to 10 nmols phosphate and diluted in 3 CHCl_3_: 2 MeOH. Lipid solutions were dried at 180 °C. Following cooling, samples were incubated with 50 ml of concentrated perchloric acid to DOPC samples, 80 ml to 0 mol% PIP_2_ samples, 100 ml to 1 mol% PIP_2_ samples, and 250 ml to 4 mol% PIP_2_ samples. Samples were heated for 20 to 45 min on a heating block (see Fig. S4 for optimization of incubation time). Following a cooling step, samples were mixed with 400 ml of MQ H_2_O, 2 ml of Ammonium Molybdate/Malachite Green working solution, and 80 ml of 1.5% Tween 20 solution. Samples were mixed and absorbance read at 660 nm. Data were acquired in triplicate.

### Microscopy

TIRF experiments were performed on a Nikon Eclipse Ti2 inverted microscope equipped with a motorized Epi/TIRF illuminator, Perfect Focus system, and a motorized stage. Imaging was performed using a 100X oil TIRF objective (numerical aperture = 1.49). Multicolor imaging used a quad-color excitation cube. A reflection interference contrast (RICM) excitation cube was used for high contrast characterization of substrates and cell membranes. Images were captured on an EM-CCD detector (iXon Lite L897, Andor Inc.). All peptide-treated samples were excited using a 405 nm laser in a TIRF configuration and emission was collected at 405 nm (405–405) and 488 nm (405–488). Exposure times, multidimensional acquisitions, and time-lapse periods were set using Nikon Elements software.

### Data analysis

All analysis was performed using functions in MATLAB equipped with the image processing toolbox DipImage. Images were background and flat-field corrected prior to making ratiometric comparisons. For background subtraction, baseline signals in the 405-405 and 405 to 488 channels, in the absence of DAN13aa, were determined on SSLBs or in cells and the average for many measurements was applied to other raw data. For images of bilayers, data acquired as image tiles were cropped to single fields of view. Ratiometric analysis was carried out by image division in a pixelwise manner. Calibrations were based on mean intensities across at least 25 fields of view in at least three independent experiments using unique stacked supported lipid bilayers.

Cell images were also acquired using a tiling strategy using RICM and fluorescent channels, as described above. Individual cells were masked using the reflection interference contrast (RICM) image. Masked data were used for ratiometric calculations.

For texture analyses, average pixel intensity in the cell mask was calculated and plotted against the gray-level co-occurrence matrix (GLCM). The GLCM contrast was implemented in MATLAB, using existing functions.

The formula for GLCM contrast is:

$$\text{Contrast}=\sum_{i=0}^{N-1}\sum_{j=0}^{N-1}{\left(i-j\right)}^{2}\;p\left(i,j\right)$$

For simulations, an empty 512 × 512 image was created, and a circle of 200 pixels was inscribed in the center to represent the cell surface. Spots were simulated as Gaussian objects with random locations, within the circle and varied in intensity and number. Poisson noise was added. Three classes of simulations were created: 1. low mean intensity, low heterogeneity, 2. low mean intensity, moderate heterogeneity, and 3. high mean intensity and high heterogeneity. For the high heterogeneity dataset, clusters of multiple points, determined using a random number generator, were independently generated. For each group of simulations, 50 iterations were run prior to processing using the same GLCM workflow as the microscopy data.

## Data availability

Data will be made available through a repository.

## Supporting information

This article contains supporting information.

## Conflict of interest

The authors declare that they have no conflicts of interest with the contents of this article.
