# Supplementary material for: Detection of PIP2 distributions in biological membranes using a peptide-based sensor
Source: J Biol Chem. 2025 Oct 16;301(12):110826. doi: 10.1016/j.jbc.2025.110826 (PMC12664033; doi:10.1016/j.jbc.2025.110826)
Supplement: Supplemental Figures [file mmc1.docx]

**Supporting Information**


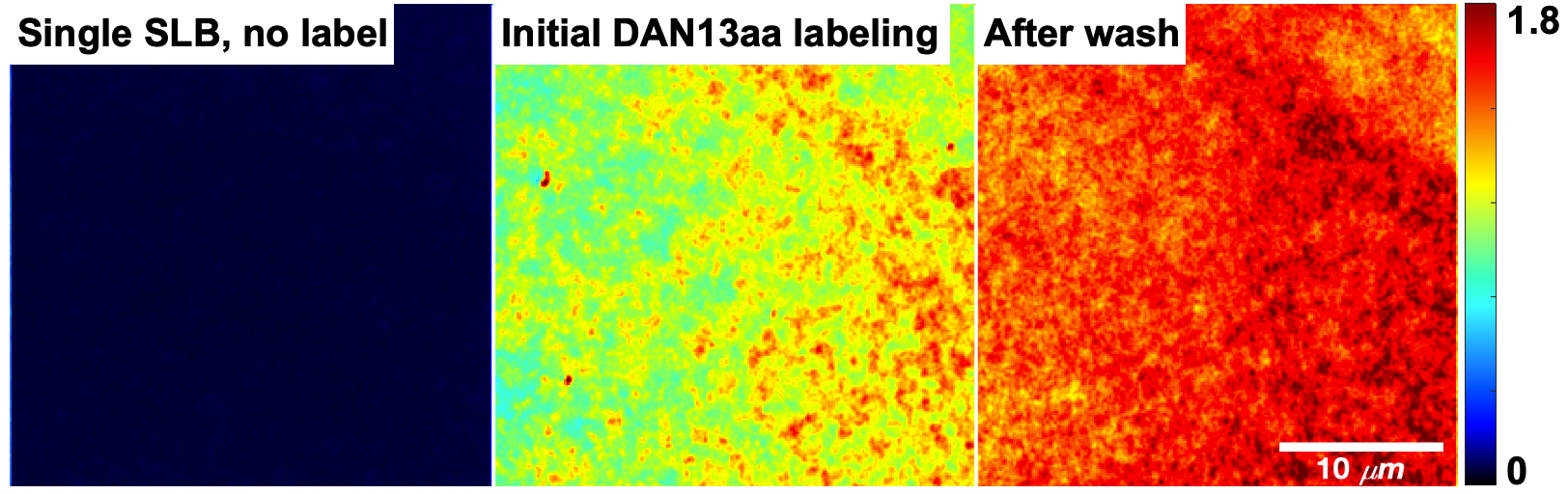


**Supplemental Figure 1**. DAN13aa sensor accumulation on glass displaying a single SLB. Representative, ratiometric snapshots of a single supported lipid bilayer on etched glass before (left), during initial exposure to DAN13aa (middle), and after washing (right), respectively, following 10 minutes of incubation with the sensor.


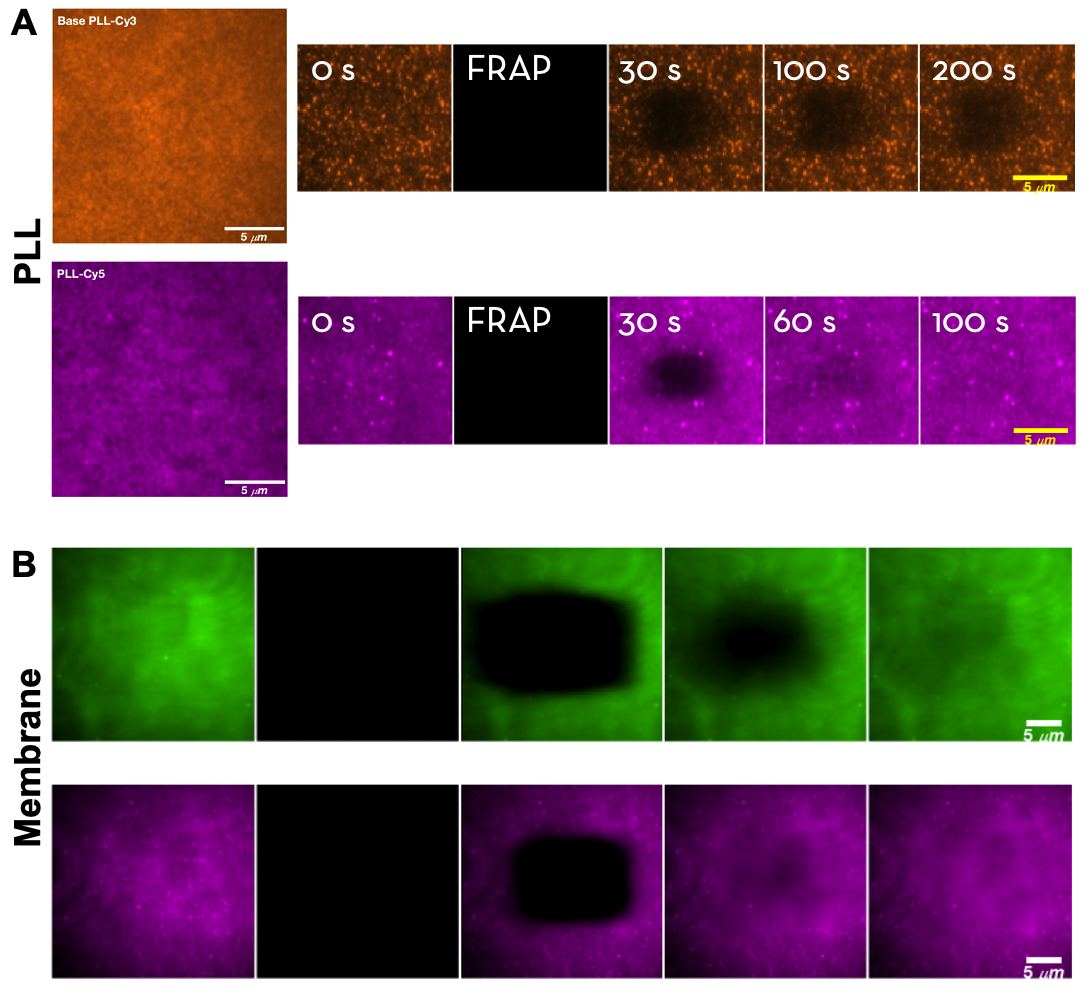


**Supplemental Figure 2**. Mobility of components of stacked supported bilayer configuration. Characterization of mobility of the components of SSLBs by fluorescence recovery after photobleaching (FRAP). A. Fluorescent poly-D-lysine (PDL-Cy3) on etched glass was immobile (top), whereas the PDL-Cy5 between the two SLBs was mobile. B. FRAP of lower SLB following deposition and labeling with DiO (top, green) and followed by deposition of PLL and upper SLB. Upper SLB FRAP following labeling with DiI (bottom, magenta).

**Supplemental Figure 3**. Single PIP_2_ diffusion in a stacked SLB configuration. PIP_2_-TopFluor was doped into the top SLB in the stacked configuration at 4 mole percent. Following bleaching down to single molecule levels, individual lipids could be tracked. Tracks are shown for 5 seconds of imaging at 20 frames per second.


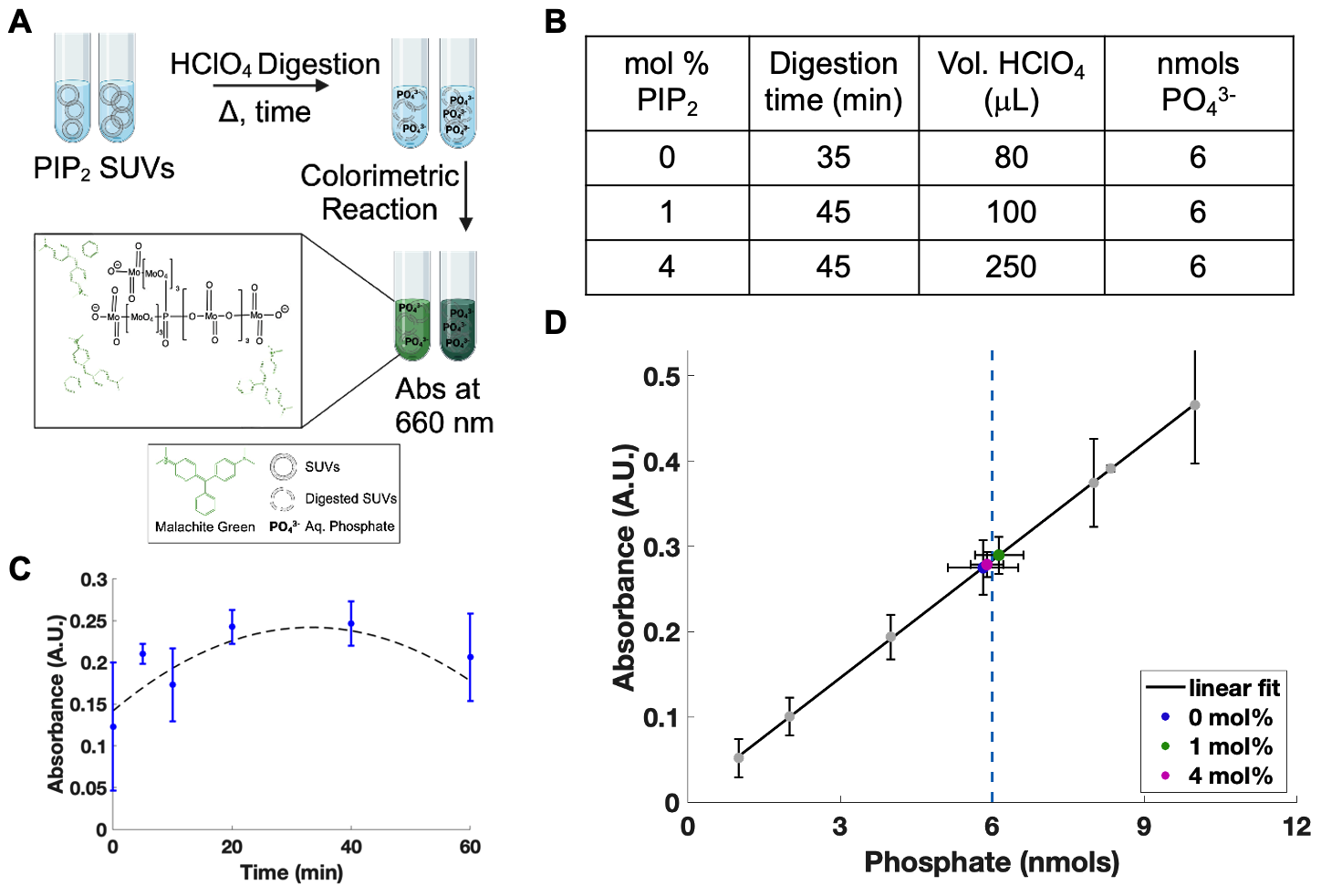


**Supplemental Figure 4**. Verification of reconstituted PI(4,5)P_2_ content in membranes using a colorimetric assay. A. Schematic of assay. Vesicles were prepared and digested with perchloric acid prior to application of the colorimetric Malachite Green reagent. B. Table of vesicle compositions and digestion times. C. Digestion times were optimized to achieve peak signal detection. D. Measurement of phosphate amounts in samples using assay. Each sample targeted a final amount of phosphate to be 6 nanomoles.


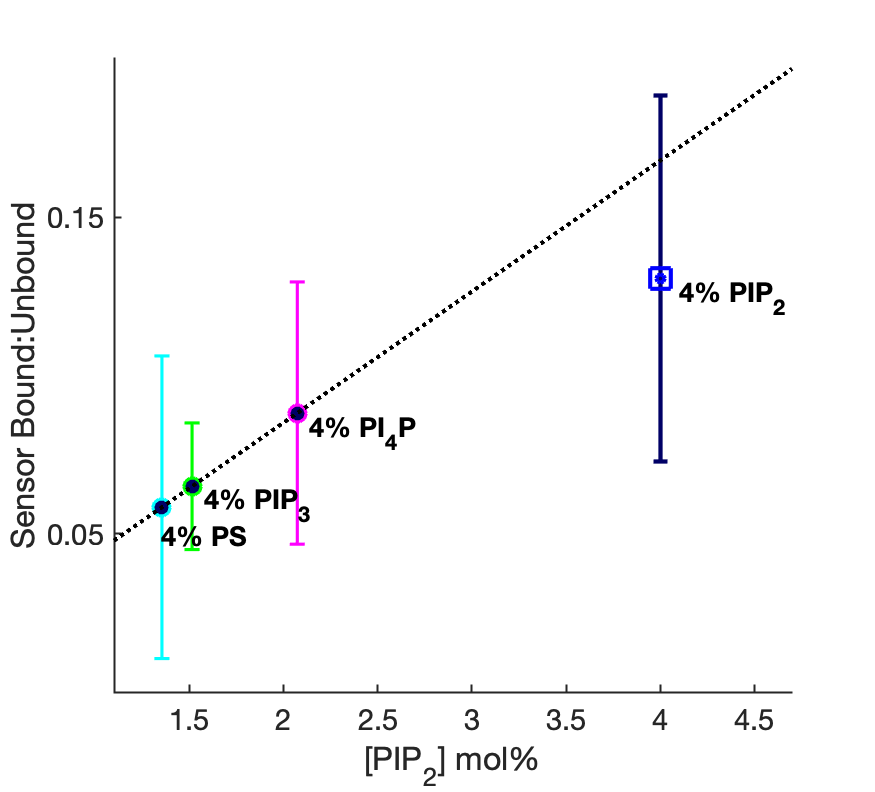


**Supplemental Figure 5**. DAN13aa sensor detection of other anionic lipids in SSLBs. Dashed line is a segment of the standard curve, as shown in Figure 2 with the corresponding data for 4 mol% PI(4,5)P_2_ (right and boxed in blue). For each of 4 mol% PI(3,4,5)P_3,_ 4 mol% PI(4)P, and 4 mol% PS, the ratiometric signal measured using the DAN13aa sensor is plotted onto the standard curve, showing the false positive signal from each non-specific lipid. Each set of data is from at least 10 fields of view and at least 3 SSLBs.


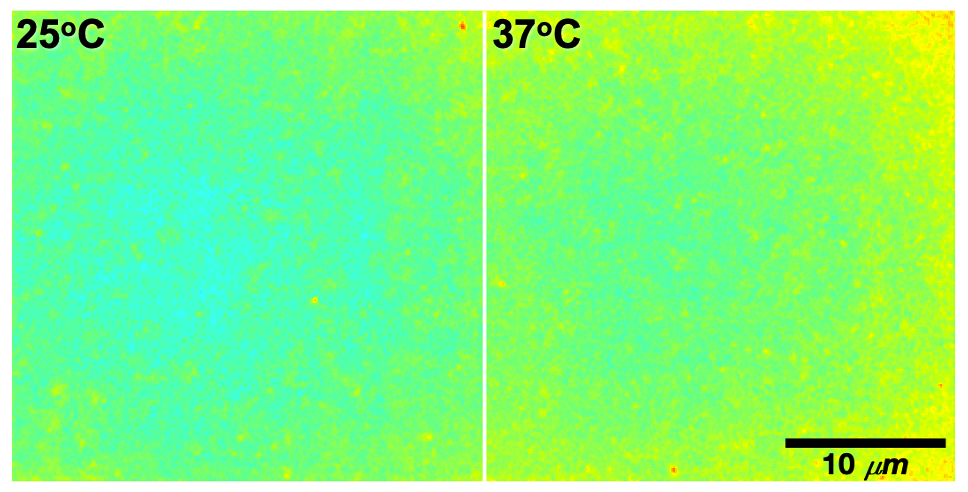


**Supplemental Figure 6**. DAN13aa sensor performance as a function of temperature. Ratiometric images for stacked SLBs imaged at either 25^o^C or 37^o^C.


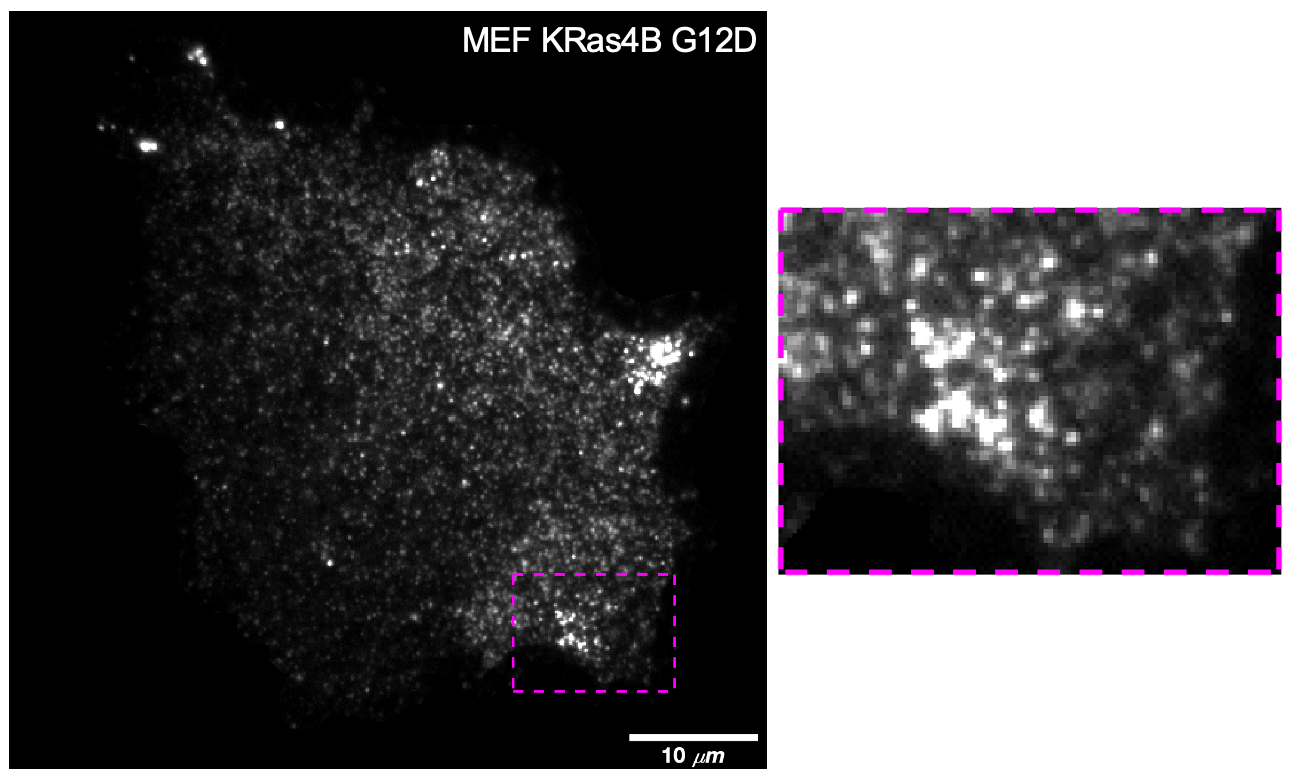


**Supplemental Figure 7**. IF and heterogeneity in mutant membranes. Representative images of MEF KRas4B G12D mutant cells stained for PI(4,5)P_2_ and imaged in TIRF. Zoomed region shows clustered lipids.

**
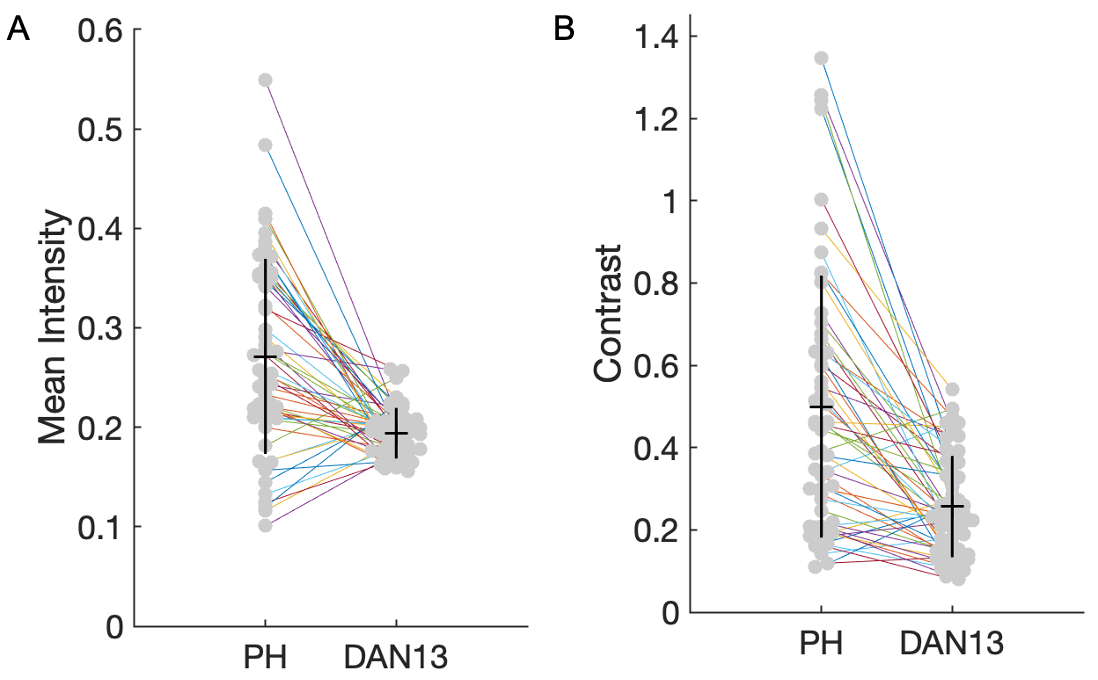
**

**Supplemental Figure 8**. Single cell distributions for MEFs expressing PH-RFP and labeled with DAN13aa. A. Mean intensity (in mole percent) of PIP_2_. B. GLCM contrast of PIP_2_ distribution. For the same single cell, the signal detected via PH domain expression and DAN13aa labeling are connected by a line. Overall spreads across the population of cells are plot as mean and standard deviation. Data are from 2 independent experiments.
